# LncRNA Xist Contributes to Endogenous Neurological Repair After Chronic Compressive Spinal Cord Injury by Promoting Angiogenesis Through the miR-32-5p/Notch-1 Axis

**DOI:** 10.3389/fcell.2020.00744

**Published:** 2020-08-06

**Authors:** Xing Cheng, Jin Xu, Zhengran Yu, Jinghui Xu, Houqing Long

**Affiliations:** ^1^Department of Spine Surgery, The First Affiliated Hospital, Sun Yat-sen University, Guangzhou, China; ^2^State Key Laboratory of Biocontrol, School of Life Sciences, Sun Yat-sen University, Guangzhou, China

**Keywords:** endogenous neurological repair, chronic compressive spinal cord injury, angiogenesis, Xist, miR-32-5p, Notch-1

## Abstract

Endogenous repair after chronic compressive spinal cord injury (CCSCI) is of great clinical interest. Ischemia-hypoxia-induced angiogenesis has been proposed to play an important role during this repair process. Emerging evidence indicates that long non-coding RNAs (lncRNAs) are involved in the pathophysiological processes of various diseases. Here, we identified a lncRNA (Xist; X-inactive specific transcript) with upregulated expression in cervical spine lesions during endogenous neurological repair in CCSCI rats. Therapeutically, the introduction of Xist to rats increased neurological function *in vivo* as assayed using the Basso, Beattie, and Bresnahan (BBB) score and inclined plane test (IPT). We found that the introduction of Xist enhanced endogenous neurological repair by promoting angiogenesis and microvessel density after CCSCI, while depletion of Xist inhibited angiogenesis and cell sprouting and migration. Mechanistically, Xist promoted angiogenesis by sponging miR-32-5p and modulating Notch-1 expression both *in vitro* and *in vivo*. These findings suggest a role of the Xist/miR-32-5p/Notch-1 axis in endogenous repair and provide a potential molecular target for the treatment of ischemia-related central nervous system (CNS) diseases.

## Introduction

Chronic compressive spinal cord injury (CCSCI) is one of the most common causes of non-traumatic incomplete spinal cord injury (SCI) in middle-aged and elderly patients (Tetreault et al., 2015). Compression-induced ischemia-hypoxia is an important pathophysiological change after CCSCI (Fehlings and Skaf, 1998; Moghaddamjou et al., 2020). In the clinic, some patients with serious CCSCI show no neurological symptoms, while those with mild CCSCI may suffer different levels of neurological impairment. It is known that endogenous neurological repair after incomplete SCI plays an important role in the variation of neurological symptoms among patients (Raineteau and Schwab, 2001; Rasmussen and Carlsen, 2016). Understanding the molecular mechanisms underlying endogenous neurological repair will facilitate the clinical treatment of CCSCI.

It has been reported that vascular events after SCI not only affect the progress of secondary tissue damage but also establish a new environment that may promote neural plasticity in the chronically injured spinal cord (Mautes et al., 2000; Wang et al., 2018). We have previously revealed that the extent of angiogenesis is related to neurological function recovery following chronic compressive SCI (Long et al., 2014; Cheng et al., 2015). Zoe et al., reported that loss of proliferating NG2 + pericytes prevents intra-lesion angiogenesis and influences scar-forming processes, impairing endogenous repair after SCI in mice (Hesp et al., 2018). A therapy that uses intrathecal anti-Nogo-A antibodies is currently in clinical trials for SCI since it may improve vascular sprouting and repair and reduce neurological deficits (Zörner and Schwab, 2010; Rust et al., 2019). This evidence confirms that angiogenesis plays a key role in the progress of neurological repair after SCI, especially during the chronic injury phase. Many factors and pathways have been shown to be important in the regulation of angiogenesis (Casella et al., 2002; Kitamura et al., 2007; Acar et al., 2012; Yang et al., 2017; Ni et al., 2019). Notch-1 is a well-known factor that can alter the level of angiogenesis in various diseases including rheumatoid arthritis (Gao et al., 2012), cancer (Dos Santos et al., 2017; Jin et al., 2017), and psoriasis (Rooney et al., 2014) by regulating downstream genes.

Long non-coding RNAs (lncRNAs) have emerged as an important factor in multiple pathophysiological processes of central nervous system (CNS) diseases, such as angiogenesis, neurogenesis, and apoptosis (Chandran et al., 2017). For instance, the lncRNA CCAT2 promotes angiogenesis in glioma through the activation of VEGFA signaling by sponging miR-424 (Sun et al., 2020). The lncRNA MALAT-1 has been shown to have neuroprotective effects on spinal cord ischemic/reperfusion injury by regulating miR-204 (Qiao et al., 2018). LncRNAs have also been reported to participate in the regulation of stem cell self-renewal and differentiation in stroke-induced neurogenesis (Fan et al., 2020). Recent studies suggest that lncRNAs are important regulators of the pathophysiological processes of CNS diseases, especially those associated with ischemia; therefore, we are interested in the contribution of lncRNAs to endogenous neurological repair after CCSCI. Some research have proposed that the neurological functional recovery of female is often better than that of male after SCI in human and rodent studies (Sipski et al., 2004; Xiao et al., 2017). Interestingly, we identified a female specific lncRNA, X-inactive specific transcript (Xist), which showed differential expression during endogenous neurological repair in CCSCI rats. Bioinformatics analysis showed that Xist and Notch-1 (a well-known factor for angiogeneiss) are direct targets of miR-32-5p. We found that Xist improved neurological function *in vivo* by promoting angiogenesis through the miR-32-5p/Notch-1 axis. We further investigated the function of the Xist/miR-32-5p/Notch-1 axis in angiogenesis during endogenous neurological repair and confirmed its role in tube formation and cell sprouting and migration *in vitro*. Our findings indicate that the Xist/miR-32-5p/Notch-1 axis may be a novel molecular target for endogenous repair in ischemia-associated CNS diseases.

## Materials and Methods

### Animal Studies

All experimental procedures were approved by the Research Ethics Committee of Sun Yat-sen University, Guangzhou, China and conformed to all relevant regulatory standards. In total, 192 adult female Sprague–Dawley rats (weighing 250–300 g; aged 28–30 weeks) were randomly allocated to the Sham (*n* = 25) and chronic compressive spinal cord injury (CCSCI) (*n* = 167) groups. According to the study duration and treatments, CCSCI rats were randomly divided into the following groups: the 4-week CCSCI group (4W SCI; *n* = 25); the 8-week CCSCI group (8W SCI; *n* = 43); the 8-week CCSCI with Xist small interfering RNA group (8W SCI + siXist; *n* = 31); the 8-week CCSCI with NC Xist siRNA group (8W SCI + NC siXist; *n* = 31); the 8-week with DAPT (8W SCI + DAPT; *n* = 8); the 8-week with NC DAPT (8W SCI + NC; *n* = 9); the 8-week with agomiR-32-5p group (8W SCI + agomiR-32-5p; *n* = 9); and the 8-week with agomiR-32-5p and Xist group (8W SCI + agomiR-32-5p + Xist; *n* = 11). The rats were housed in groups of 4-5 per cage on a 12/12-h light/dark cycle with free access to food and water.

### Intraperitoneal/Intravenous Injection and Gene Delivery

DAPT solution (1 μg/μL) was prepared by dissolving DAPT powder (MCE, United States) in 0.01 M phosphate-buffered saline (PBS) containing 5% dimethyl sulfoxide. For gene delivery, 10 μL recombinant lentivirus (GeneCopoeia, Guangzhou, China) was delivered to the rats using a microinjection syringe via intraperitoneal or intravenous injection 4 wpi. Solutions at 5 × 10^7^ titer units (TU)/mL containing 50 μL negative control, siXist, or Xist lentivirus (3 μL/g per rat), DAPT (1 mg/kg per rat) solution, and agomiR-32-5p (3.5 μL/g per rat) were used. AgomiR-32-5p was synthesized by BGI (Shenzhen, China). MiR-32-5p mimics and inhibitors were purchased from Guangzhou Yeshan Biological Technology Co., Ltd. The sequences are shown in Supplementary Table S1. HUVECs were transfected with miR-32-5p mimics, inhibitors, or their parental negative controls using Lipofectamine^®^ 2000 reagent according to the manufacturer’s instructions (Invitrogen).

### Chronic Compressive Spinal Cord Injury

Each rat in the sham and CCSCI groups was anesthetized with 10% chloral hydrate (300 mg/kg) (Guangzhou FISCLAB Environ. Sci-Tech. Co., Ltd., Guangzhou, China).

Following exposure of the spinal process and laminas of C4-C6 from the posterior, the ligamentum flavum and C5 lamina were removed to access the epidural space. In the SCI group, the polymer (1→4)-3,6-anhydro-a-l-galactopyranosyl-(1→3)-β-D-galactopyranan) sheet (1 mm × 3 mm × 1 mm) was implanted into the C6 epidural space on the dorsal part of the spinal cord. Spinal cord compression was achieved by expansion of the polymer caused by liquid absorption (Long et al., 2014; Cheng et al., 2015). This sheet can absorb liquid in the spinal canal to expand its volume sevenfold (around 2.3 mm × 4.2 mm × 2.2 mm) at the end (Kim et al., 2004; Long et al., 2013). In the sham group, the C5 lamina was removed without insertion of the polymer. Following surgery, the incision was closed in layers with complete hemostasis. To prevent dehydration, animals received a subcutaneous (s.c.) injection of lactated Ringer’s solution (200 μL) immediately after surgery. All rats were administered an intramuscular injection of penicillin G (80 U/g) during surgery to prevent infection, and carprofen (4–5 mg/kg, Rimadyl, Pfizer) was injected subcutaneously 2 days post-surgery for further pain relief as needed. All surgeries were performed by the same experienced investigator.

### Neurological Function Evaluation

To evaluate the recovery of neurological function after CCSCI, motor function was assessed using the Basso, Beattie, and Bresnahan (BBB) locomotor scale (Basso et al., 1995) and inclined plane test (IPT) (Chang et al., 2008) on day 1 post-injury (dpi) and 1, 2, 3, 4, 5, 6, 7, and 8 weeks post-injury (wpi). The range of BBB scores was between 0 and 21. A score of 0 reflects complete paralysis, whereas a score of 21 indicates normal locomotion. Lower scores (0–7) express isolated joint movements with little or no hindlimb movement; intermediate scores (8–13) express intervals of uncoordinated stepping; and higher scores (14–21) express forelimb and hindlimb coordination. For the IPT, rats were placed horizontally on a smooth tilt board. The angle of the board started from a horizontal position (0°) and increased by 5–10° after every attempt. The maximum angle at which the rats remained on the board for 10 s was recorded. BBB score raw data in the 8W SCI group were used to evaluate the recovery of animals at 8 wpi as compared with at 4 wpi [Recovery BBB score (SCI): BBB score (8 wpi) – BBB score (4 wpi); Recovery BBB score ≥ SD of recovery BBB score (SCI) was defined as significantly recovered, and no recovery was defined as a recovery BBB score < SD of the recovery BBB score (SCI)]. The evaluation was conducted by two investigators blinded to the group assignments.

### Micro-Computed Tomography

To assess the alteration in microvessel density in the lesion side of the cervical spinal cord, the cervical spinal cord of rats in different groups was subjected to micro-computed tomography (Micro-CT) (ZKKS-MCT-Sharp-II, Guangzhou Zhongkekaisheng Medical Technology Co., Ltd., Guangzhou, China) scanning after gelatin-lead oxide intracardiac injection. Animals were anesthetized with an overdose of 80–120 mg/kg intravenous sodium pentobarbital (Guangzhou Fischer Chemical Co., Ltd) and transcardially perfused using heparinized saline by adjusting the perfusion height (110 cm H_2_O) and rate (20 mL/min). Subsequently, gelatin-lead oxide mixed liquor (medical gelatin 5 g, lead oxide 100 g, distilled water 100 mL) was continually infused into the aortic cannula by syringe (6–8 mL/min) for 5 min, until the viscera was completely red, to ensure full diffusion of the contrast medium. The cervical spinal cords were then carefully harvested and fixed overnight with 4% formaldehyde in phosphate-buffered solution. Prior to micro-CT scanning, the tissues surrounding the spine were carefully removed under a microscope. Three-dimensional reconstruction of each specimen was performed using the 3D-Med 4.3 software (Guangzhou ZhongkeKaisheng Medical Technology Co., Ltd., Guangzhou, China) and the relative microvessel density of all specimens in each group was calculated.

### Immunohistochemistry

In brief, rats in different groups were euthanized with an overdose of intravenous sodium pentobarbital and transcardially perfused with 0.9% saline followed by 4% paraformaldehyde in 0.1 M phosphate buffer (PFA). The C5-C7 spinal cord was harvested, fixed overnight with 4% formaldehyde in phosphate-buffered solution at 4°C, and embedded in paraffin. A series of 25-μm thick spinal cord sections were used for immunohistochemical staining. Slides were placed in a plastic rack and vessel for microwave epitope retrieval. Sections were incubated overnight at 4°C with a rabbit polyclonal antibody against Notch-1 (1:200; ab8925, Abcam, Cambridge, United Kingdom), a mouse monoclonal antibody against HES-1 (1:50; sc-166410, Santa Cruz Biotechnology, Inc., United States), and a mouse monoclonal antibody against VEGFA (1:200; ab1316, Abcam, Cambridge, United Kingdom). Subsequently, the sections were sequentially incubated with a ready-to-use DAKO ChemMate EnVisionTM kit (cat. no. K500711; Dako; Agilent Technologies, Inc., Santa Clara, CA, United States) for 30 min at room temperature. Images of each section of the perilesional spinal cord were taken at 20×, 200×, and 400× magnification using an XC30 camera mounted on an Olympus microscope (Olympus Corporation, Tokyo, Japan). Three random images at 200 × magnification were captured for semi-quantitative analysis. The brown area in the sham spinal cord was analyzed and background density was excluded to create the standard. Subsequently, the integrated optical density (IOD) was calculated using the Image-Pro Plus 6.0 software (Media Cybernetics, Inc., Rockville, MD, United States).

### *In situ* Hybridization

*In situ* hybridization (ISH) of HIF-1α was performed using a commercial kit (Wuhan Boster Bio., Co., Ltd) to evaluate the level of hypoxia at 4 wpi and 8 wpi. Briefly, spinal cord sections (4-μm) were dewaxed, dehydrated, and washed. Subsequently, endogenous peroxidase was blocked with 3% H_2_O_2_ and sections were pretreated with antigen retrieval using proteinase K (1 μg/mL) in citromalic acid (1 mol/L) at 37°C for 2 h. After prehybridization, the sections were hybridized using HIF-1α oligonucleotide probes (Wuhan Boster Bio., Co., Ltd) at 42°C overnight. The hybridization solution was removed by 2 × saline sodium citrate (SSC) at 37°C for 10 min, 1 × SSC twice at 37°C for 5 min, and 0.5 × SSC for 10 min at room temperature. Sections were then incubated with blocking solution (5% BSA in 1% PBST) at room temperature for 1 h. Subsequently, sections were incubated for 1 h at 37°C with biotinylated mouse anti-digoxin (Wuhan Boster Bio., Co., Ltd) followed by the addition of SABC solution (Wuhan Boster Bio., Co., Ltd) at 37°C for 20 min. A DAB chromogenic substrate kit was used and color development was controlled under a microscope within 30 minutes. Section were counterstained with Mayor’s hematoxylin and dehydrated. Images of each section of the spinal cord were taken at 100× and 400× magnification using an XC30 camera mounted on an Olympus microscope (Olympus Corporation, Tokyo, Japan). The cytoplasm staining of positive cells is brownish yellow.

### Cell Culture

For *in vitro* experiments, human umbilical vein endothelial cells (HUVECs) and human embryonic kidney (HEK) 293T cells were purchased from the American Type Culture Collection (Manassas, VA, United States). Cells were cultured in Endothelial Cell Growth Medium (Merck, Darmstadt, Germany) supplemented with 10% heat-inactivated fetal bovine serum (FBS; Thermo Fisher Scientific, 10500-064) and 1% penicillin/streptomycin (Sigma, St. Louis, MO, United States). HEK-293T cells were cultured in Dulbecco’s Modified Eagle Medium (DMEM; Gibco, Grand Island, NY, United States) supplemented with 10% FBS. HUVECs were seeded at an appropriate number on an appropriate size of culture flask (Sarstedt AG & Co., Nümbrecht, Germany). Cells were maintained in an incubator filled with 95% air and 5% CO_2_ at 37°C. To simulate the hypoxic-ischemic conditions caused by CCSCI, HUVECs were incubated in a hypoxic incubator filled with 94% N_2_, 5% CO_2_, and 1% O_2_ at 37°C, as previously described (Hu et al., 2019). There were 9 groups for *in vitro* experiments as follows: Control; Hypoxia; Hypoxia transfected with NC Xist siRNA (H + NC siXist); Hypoxia transfected with Xist siRNA (H + siXist); Hypoxia transfected with NC miR-32-5p (H + NC miR-32-5p); Hypoxia transfected with miR-32-5p mimics (H + miR-32-5p mimics); Hypoxia transfected with miR-32-5p mimics and Xist (H + miR-32-5p mimics + Xist); Hypoxia with DAPT treatment (H + DAPT); and Hypoxia with DAPT treatment and transfected with miR-32-5p inhibitors (H + DAPT + miR-32-5p inhibitors).

### Tube Formation Assay

To evaluate the angiogenic capacity *in vitro*, HUVECs were harvested and seeded on Matrigel-coated 48-well plates (BD Biosciences) at a density of 2 × 10^4^/well and incubated under different conditions (normal and hypoxia). Following different treatments for 48 h, tube formation was visualized and photographed using a phase contrast inverted microscope at 100 × magnification. Quantitation of the number of tubules was performed using the Image J software with the Angiogenesis Analyzer plugin16.

### Sprouting Assay

To evaluate the sprouting capacity, a modified assay was performed as described previously (Hu et al., 2019). HUVEC monolayers were digested with 0.25% trypsin and seeded on a prepared 6-well plate in M199 medium containing 10% FBS. HUVECs were grown in clumps as observed under a microscope, following which the cell clusters were incubated with VEGF gel on a 24-well plate under different conditions. After 48 h, the total number of sprouts on the 5 cell clusters per well in each group was recorded and averaged.

### Wound Healing Assay

To evaluate the migration capacity, the bottom of plates containing HUVECs at 90% confluence was scratched with a 1000-μL pipette tip to create a linear region void of cells. The media was then removed and the cells were washed with PBS, subjected to the appropriate treatment, and incubated under different conditions. Images of the denuded zone were recorded using an inverted microscope (Olympus, Japan) at 0 and 48 h. Data are representative of three individual experiments.

### Real-Time Quantitative Polymerase Chain Reaction

Total RNA was extracted from the cervical spinal cord and HUVECs using TRIzol^®^ regent (Invitrogen, Carlsbad, CA, United States) according to the manufacturer’s protocol. The purity of the RNA was determined and reverse transcription (PrimeScriptTM RT reagent Kit) and real-time quantitative PCR (SYBR Premix Ex Taq II) were performed following the kit instructions (Takara Biotechnology Co., Ltd., China). The detailed primer sequences used in RT-qPCR are exhibited in Supplementary Table S2. Threshold cycle (CT) values were recorded and the relative expression of target genes was calculated using the equation 2^–ΔΔCt^ (Hu et al., 2019).

### Luciferase Reporter Assay

The Xist fragment containing the putative miR-32-5p binding site was cloned into the pmirGlo vector (Promega, United States) and named WT Xist. Mutation of the putative miR-32-5p target sequence in the 3-UTR of Xist was generated using a site-directed gene mutagenesis kit (Takara) and named MUT Xist. HEK-293T cells were co-transfected separately with the two constructs or empty vector and miR-32-5p mimics or inhibitors. For miR-32-5p and Notch-1 interaction, the putative and mutated miR-32-5p target binding sequences in Notch-1 were synthesized and cells were co-transfected with the WT or MUT Notch-1 reporter gene plasmid or pmirGlo plasmids using Lipofectamine^®^ 2000 (Invitrogen). At 48 h post-transfection, cells were harvested and the firefly luciferase activity was measured using a dual-luciferase reporter assay system (Promega) and normalized to Renilla luciferase activity (Jin et al., 2018; Hu et al., 2019).

### Statistical Analysis

All data are expressed as the mean ± standard error of the mean (SEM). Statistical tests were carried out using the GraphPad Prism 7.0 software (San Diego, CA, United States). Motor function recovery results were analyzed by repeated measures analysis of variance (ANOVA) to reveal overall group differences and significant changes over time. A Student’s *t*-test was performed for comparisons between two groups, and one-way analysis of variance followed by Tukey’s *post hoc* test was used for comparisons among multiple groups. Statistical significance was set at *p* < 0.05.

## Results

### Xist Promotes Endogenous Neurological Repair After CCSCI

**FIGURE 1:**
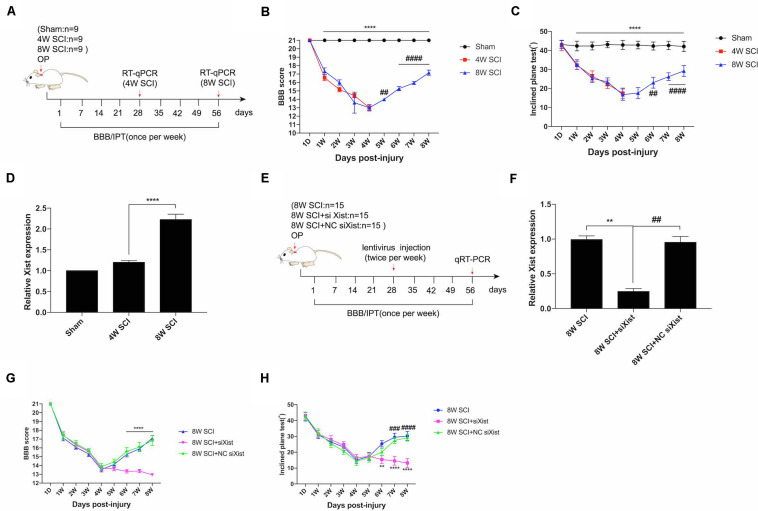
Xist is upregulated during endogenous neurological repair after CCSCI. **(A)** Experimental design. **(B)** Animals were tested weekly starting from 1 dpi in an open field, and the endogenous neurological repair was evaluated according to the Basso, Beattie, and Bresnahan (BBB) score. ****p < 0.0001: the 8W SCI group vs. the sham group; ^##^*p* < 0.01, ^####^*p* < 0.0001: the 8W SCI group vs. the 4W SCI group. **(C)** Animals were tested weekly starting from 1 dpi using the inclined plane test (IPT), and the angle (°) was recorded to reveal endogenous neurological repair. ****p < 0.0001: the 8W SCI group vs. the sham group; ^##^*p* < 0.01, ^####^*p* < 0.0001: the 8W SCI group vs. the 4W SCI group. **(D)** Relative Xist expression in the cervical spinal cord was determined by RT-qPCR in the sham, 4W SCI, and 8W SCI groups. ****p < 0.0001: the 8W SCI group vs. the 4W SCI group. **(E)** Experimental design to explore the role of Xist in endogenous neurological repair. **(F)** Relative Xist expression in the cervical spinal cord was determined by RT-qPCR in the 8W SCI, 8W SCI + siXist, and 8W SCI + NC siXist groups. **p < 0.01: the 8W SCI group vs. the 8W SCI + siXist group; ^##^*p* < 0.01: the 8W SCI + siXist group vs. the 8W SCI + NC siXist group. **(G)** The endogenous neurological repair was evaluated using the BBB score. ****p < 0.0001: the 8W SCI + siXist group vs. the 8W SCI + NC siXist group. **(H)** The endogenous neurological repair was scored using the IPT. **p < 0.01, ****p < 0.0001: the 8W SCI + siXist group vs. the 8W SCI group; ^###^*p* < 0.001, ^####^*p* < 0.0001: the 8W SCI + siXist group vs. the 8W SCI + NC siXist group. Data are expressed as the mean ± SEM.

Following incomplete chronic compressive spinal cord injury (CCSCI), endogenous neurological repair occurs in a sizable number of patients; similar results have also been observed in animal models (Rosenzweig et al., 2010; Hilton et al., 2016; Rasmussen and Carlsen, 2016). Here, we used the Basso, Beattie, and Bresnahan (BBB) score and inclined plane test (IPT) to evaluate the changes in neurological function in a rat model of CCSCI. In our model, over 80% of rats significantly recovered from 4 wpi to 8 wpi (Supplementary Figure S1). Functional assays showed that the motor function gradually decreased from 1 wpi to 4 wpi and recovered from 4 wpi to 8 wpi (Figures 1A–C). The improvement in BBB and IPT scores at 8 wpi revealed that endogenous neurological repair was achieved. Interestingly, we found that the lncRNA Xist was markedly upregulated at 8 wpi (Figure 1D). To understand the role of Xist in endogenous neurological repair, we introduced siXist or its NC into CCSCI rats (Figure 1E) and examined the depletion effect using RT-qPCR (Figure 1F). As shown in Figures 1G,H, silencing Xist inhibited endogenous neurological repair. The neurological function, as indicated by the BBB and IPT scores, was not recovered from 4 wpi to 8 wpi following Xist depletion, indicating that Xist contributes to endogenous neurological repair.

### Xist Promotes Angiogenesis After CCSCI *in vivo*

**FIGURE 2:**
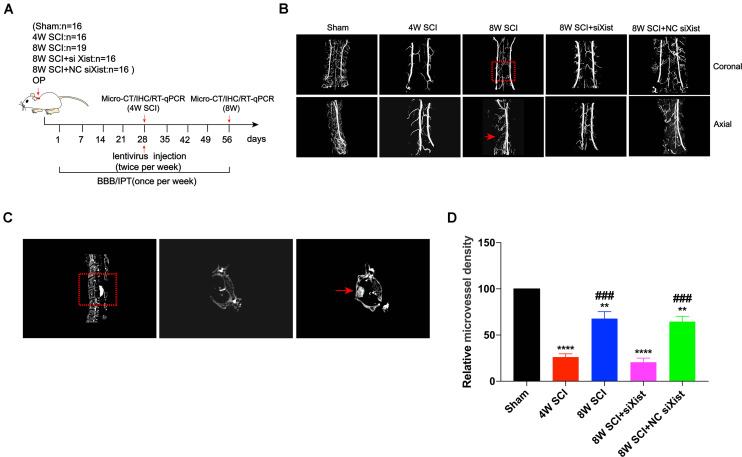
Xist knockdown inhibits angiogenesis after CCSCI. **(A)** Experimental design. **(B)** Three-dimensional reconstruction and representative micro-CT images of microvessels of the cervical spinal cord in the sham and SCI groups. The red arrow indicates the compression site of the cervical spinal cord. **(C)** Representative sagittal and transverse CT images of the resected lamina of C5 and the compression material, respectively (red arrow). **(D)** Angiogenesis was evaluated by quantitation of the relative microvessel density. **p < 0.01, ****p < 0.0001 as compared with the sham group; ^###^*p* < 0.001 as compared with the 4W SCI group. Data are expressed as the mean ± SEM.

It has been reported that angiogenesis is an important process for endogenous spinal cord repair (Casella et al., 2002). To explore the biological changes after CCSCI and the effects of Xist on endogenous neurological repair, we measured angiogenesis after CCSCI using micro-CT (Figure 2A) and vascular endothelial growth factor (VEGF) staining (Supplementary Figure S4A) for different interventions. The compression material was clearly shown on CT, verifying spinal cord compression (Figure 2C). After three-dimensional reconstruction of cervical spinal cord vessels, we found that the relative microvessel density was significantly decreased at 4 wpi (Figure 2D). The poor microvessel density of the cervical spinal cord indicated that a severe ischemia-hypoxia phase may occur at 4 wpi, as detected by assessing the expression level of HIF-1α (Supplementary Figure S2). These results are consistent with those reported in our previous study (Cheng et al., 2019). However, the relative microvessel density of the spinal cord was ameliorated at 8 wpi, accompanied by an improvement in motor function (Supplementary Figure S3). Knocking down the expression of Xist significantly decreased the microvessel density of the spinal cord as compared with the control or NC siXist at 8 wpi. No difference was seen at 4 wpi (Figures 2B,D). In addition, using VEGF as a marker of angiogenesis (Leung et al., 1989; Zeng et al., 2005), we found that depletion of Xist also significantly decreased VEGF expression (Supplementary Figure S4B). Taken together, these data indicate that the upregulation of Xist can promote angiogenesis and further improve endogenous neurological repair and motor function.

### Notch-1 and Hes-1 Expression Are Downregulated After Xist Knockdown in CCSCI

**FIGURE 3:**
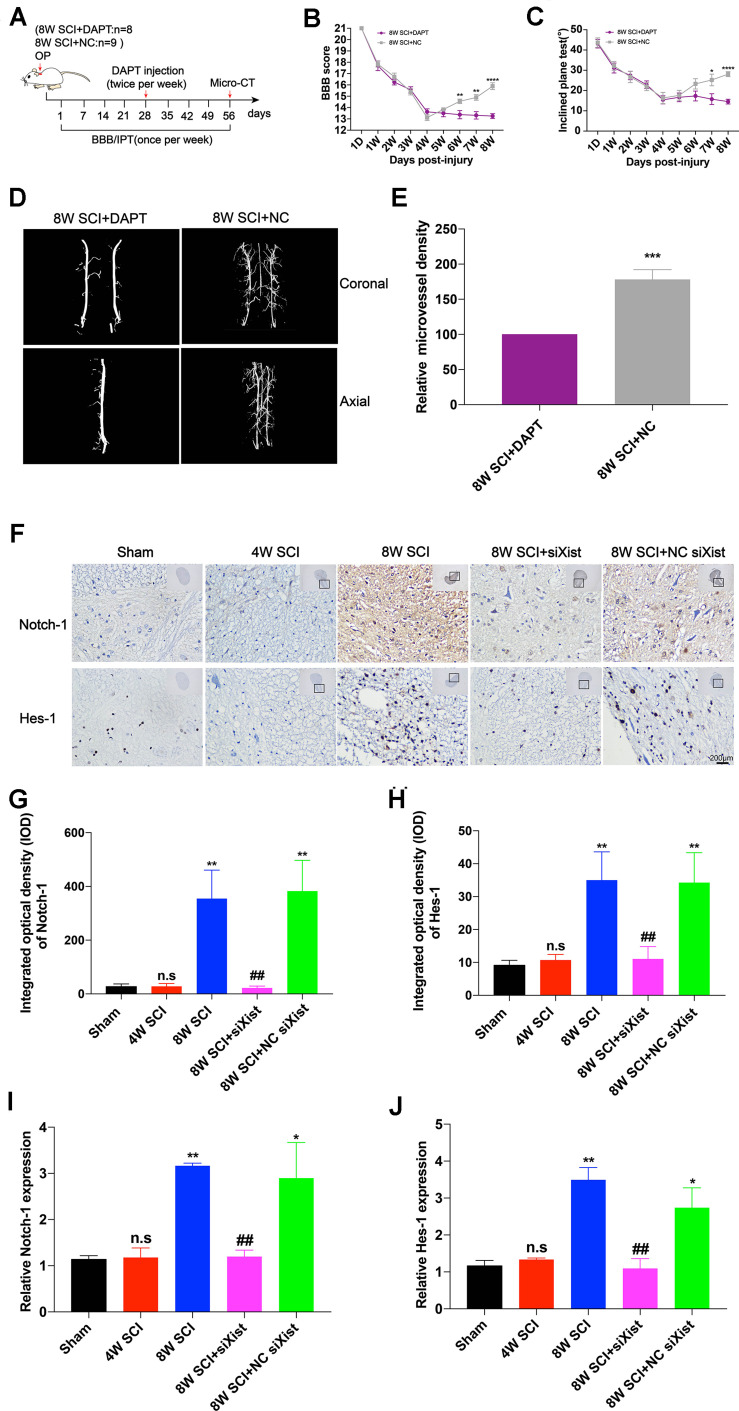
Notch-1 promotes angiogenesis and endogenous neurological repair and causes downregulation of Notch-1 and Hes-1 after Xist depletion in CCSCI rats. **(A)** Experimental design. **(B)** Neurological function was evaluated using the BBB score. **(C)** Neurological function was evaluated using the IPT. **(D)** Three-dimensional reconstruction and representative micro-CT images of the microvessels of the cervical spinal cord. **(E)** Quantitation of the relative microvessel density. Data are expressed as the mean ± SEM. *p < 0.05, **p < 0.01, ****p < 0.0001. **(F)** Notch-1 and Hes-1 expression was assessed by immunohistochemistry (20 × and 400 × magnification; scale bar, 200 μm). Notch-1 and Hes-1 are mainly expressed in the lesion site of the cervical spinal cord. **(G)** Notch-1 immunohistochemical staining was quantitated by analyzing the integrated optical density (IOD). **(H)** Hes-1 immunohistochemical staining was quantitated by analyzing the integrated optical density (IOD). **(I)** The relative Notch-1 expression was assessed by RT-qPCR. **(J)** The relative Hes-1 expression was assessed by RT-qPCR. **(G–J)** ns (non-significant) as compared with the sham group; *p < 0.05, **p < 0.01 as compared with the 4W SCI group; ^##^*p* < 0.01 as compared with the 8W SCI group. All data are expressed as the mean ± SEM. *p < 0.05, **p < 0.01, ***p < 0.001, ****p < 0.0001 as compared with 8W SCI + DAPT group.

It has been reported that Notch-1 is an important regulatory factor of angiogenesis (Gao et al., 2012). We confirmed that Notch-1 promoted angiogenesis and endogenous neurological repair after CCSCI by applying DAPT, a γ-secretase inhibitor, to deactivate the Notch-1/Hes-1 signaling pathway (Figure 3A). Figures 3B,C clearly show that neurological function was inhibited following injection of DAPT since endogenous neurological repair was not achieved at 8 wpi as compared with the control group. Further, we evaluated the alteration in microvessel density by micro-CT and found an increase in the control group at 8 wpi as expected (Figure 3D). On the contrary, following DAPT treatment, the relative microvessel density was significantly reduced as compared with the control group (Figure 3E). Moreover, VEGF immunostaining revealed that DAPT treatment decreased angiogenesis at 8 wpi following CCSCI (Supplementary Figure S5). These results confirm that Notch-1 plays a positive role in angiogenesis and endogenous neurological repair following CCSCI.

To elucidate whether Xist promotes angiogenesis through the Notch-1 pathway, we further detected the expression of Notch-1 and its downstream gene Hes-1 during CCSCI by immunohistochemistry following depletion of Xist (Figure 3F). We found that Notch-1 and Hes-1 expression in the cervical spinal cord were increased at 8 wpi in the 8W SCI group but significantly decreased following Xist depletion (Figures 3G–J). In addition, both Notch-1 and Hes-1 were mainly expressed in the compressive lesion site of the cervical spinal cord (Figure 3F). Similarly, RT-qPCR showed that the expression levels of Notch-1 and Hes-1 mRNA were significantly decreased after Xist depletion as compared with the 8W SCI and 8W SCI + NC siXist groups. The mRNA expression levels of Notch-1 and Hes-1 were no different between the 4W SCI and 8W SCI + siXist groups. These results indicate that Xist may promote angiogenesis and endogenous neurological repair by moderating Notch-1.

### Xist and Notch-1 Are Direct Targets of miR-32-5p

**FIGURE 4:**
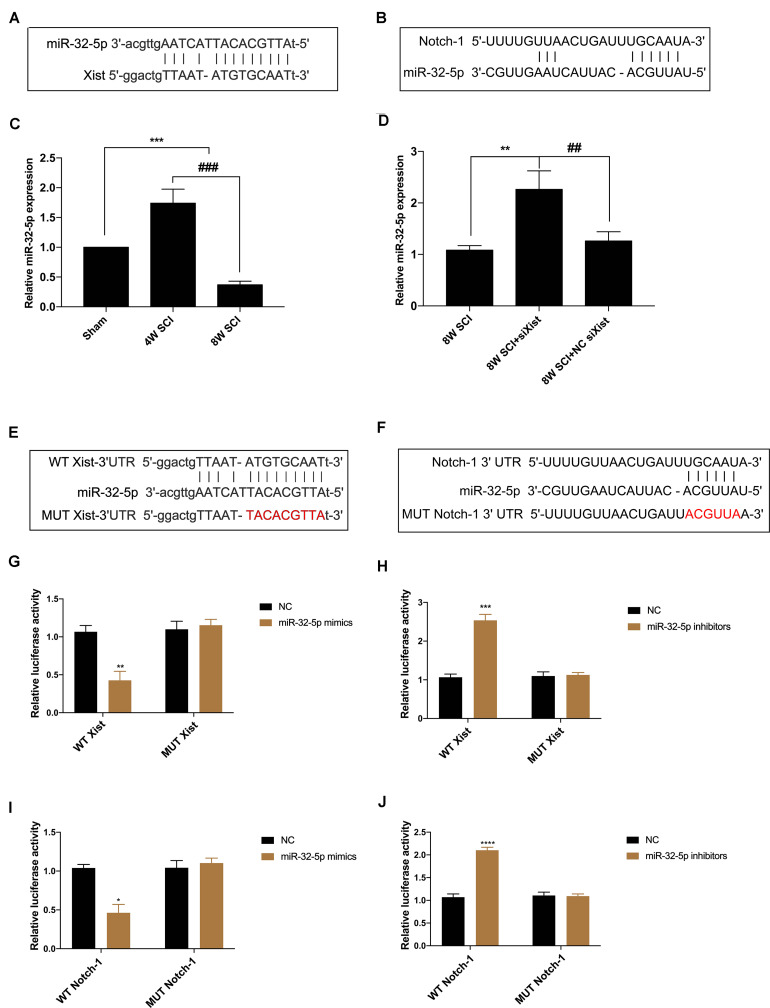
Xist and Notch-1 are direct targets of miR-32-5p. **(A)** Binding regions between miR-32-5p and Xist. Bioinformatics analysis (ChipBase, LncRNAdb, and StarBase) was used to predict the target microRNA regulated by Xist. **(B)** Binding regions between Notch-1 and miR-32-5p. Bioinformatics analysis (TargetScan, Starbase, miRanda, and miRDB) was used to predict the putative target of miR-32-5p. **(C)** Quantitation of the relative miR-32-5p expression in the sham, 4W SCI, and 8W SCI groups by RT-qPCR. **(D)** Quantitation of the relative miR-32-5p expression in the 8W SCI, 8W SCI + siXist, and 8W SCI + NC siXist groups by RT-qPCR. **(E)** Dual-luciferase reporter constructs containing the wild-type (WT Xist) or mutant (MUT Xist) Xist sequence. **(F)** Dual-luciferase reporter constructs containing the wild-type (WT Notch-1) or mutant (MUT Notch-1) Notch-1 sequence. **(G)** Dual-luciferase reporter assay of miR-32-5p and Xist following transfection of miR-32-5p mimics. HEK-293T cells were co-transfected with the miR-32-5p mimics or corresponding NC and the dual-luciferase vector containing WT Xist or MUT Xist and incubated for 48 h. **(H)** Dual-luciferase reporter assay of miR-32-5p and Xist following transfection with miR-32-5p inhibitors. HEK-293T cells were co-transfected with the miR-32-5p inhibitors or the corresponding NC and the dual-luciferase vector containing WT Xist or MUT Xist. **(I)** Dual-luciferase reporter assay of Notch-1 and miR-32-5p following transfection of miR-32-5p mimics. HEK-293T cells were co-transfected with the miR-32-5p mimics or the corresponding NC and the dual-luciferase vector containing WT Notch-1 or MUT Notch-1 and incubated for 48 h. **(J)** Dual-luciferase reporter assay of miR-32-5p and Xist following transfection with miR-32-5p inhibitors. HEK-293T cells were co-transfected with miR-32-5p inhibitors or the corresponding NC and the dual-luciferase vector containing WT Xist or MUT Xist. Three independent experiments were carried out. *p < 0.05, **p < 0.01, ***p < 0.001. ****p < 0.0001; ^##^*p* < 0.01, ^###^*p* < 0.001. Data are expressed as the mean ± SEM.

To further explore the relationship between Xist and Notch-1, we identified both Xist and Notch-1 as target genes of miR-32-5p by bioinformatics analysis (Figures 4A,B). MiR-32-5p has been reported to play a role in various biological processes such as mediating neuroinflammation and neuropathic pain and promoting cell proliferation. In addition, we observed that miR-32-5p overexpression was increased at 4 wpi and significantly decreased at 8 wpi following CCSCI (Figure 4C). Depletion of Xist upregulated the expression of miR-32-5p as compared with the control group (Figure 4D), suggesting a possible interaction between Xist and miR-32-5p. To further validate the regulatory relationship between Xist, Notch-1, and miR-32-5p, we created wild-type and mutant dual-luciferase Xist and Notch-1 constructs and expressed them in HEK-293T cells (Figures 4E,F). The dual-luciferase reporter assay showed that miR-32-5p mimics suppressed the luciferase activity of wild-type Xist but had no effect on the luciferase activity of mutant Xist (Figure 4G). Similarly, miR-32-5p inhibitors increased the luciferase activity of wild-type Xist but had no effect on the luciferase activity of mutant Xist (Figure 4H). Moreover, co-transfection of miR-32-5p mimics and the luciferase vector containing wild-type Notch-1 downregulated the relative luciferase activity in HEK-293T cells (Figure 4I), while co-transfection of miR-32-5p inhibitors and the luciferase vector containing wild-type Notch-1 increased the relative luciferase activity in HEK-293T cells (Figure 4J). These results show that Xist and Notch-1 are direct targets of miR-32-5p *in vitro*.

### Overexpression of miR-32-5p Inhibits Endogenous Neurological Repair and Angiogenesis After CCSCI

**FIGURE 5:**
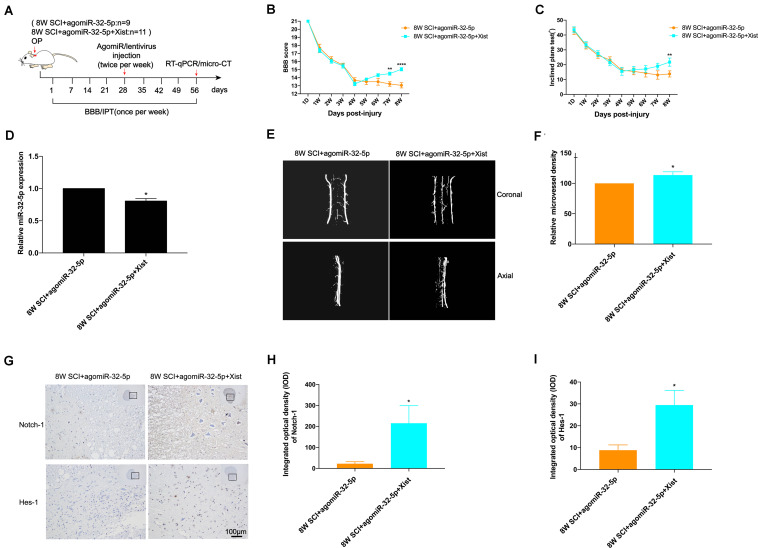
Overexpression of miR-32-5p inhibits endogenous neurological repair and angiogenesis after CCSCI by decreasing Notch-1. **(A)** Experimental design. **(B)** Neurological function was evaluated by the BBB score. **(C)** Neurological function was evaluated by the IPT. **(D)** Quantitation of the relative miR-32-5p expression. **(E)** Three-dimensional reconstruction and representative micro-CT images of the microvessels of the cervical spinal cord. **(F)** Quantitation of the relative microvessel density. **(G)** Notch-1 and Hes-1 expression was assessed by immunohistochemistry (20 × and 200 × magnification; scale bar, 100 μm). **(H)** Notch-1 immunohistochemical staining was quantitated by analyzing the integrated optical density (IOD). **(I)** Hes-1 immunohistochemical staining was quantitated by analyzing the integrated optical density (IOD). *p < 0.05, **p < 0.01, ****p < 0.0001. Data are expressed as the mean ± SEM.

To explore the function of miR-32-5p and its interaction with Xist *in vivo*, we introduced agomiR-32-5p or agomiR-32-5p and Xist into rats at 4 wpi following CCSCI (Figure 5A). We found that rats exposed to agomiR-32-5p showed almost no endogenous neurological repair, while those exposed to agomiR-32-5p and Xist showed an improvement in neurological function (Figures 5B,C). The difference in miR-32-5p expression at 8 wpi was confirmed by RT-qPCR (Figure 5D).

We further investigated angiogenesis at 8 wpi after CCSCI by micro-CT (Figures 5E,F) and VEFG immunostaining (Supplementary Figure S6A). Figure 5E show that the overexpression of miR-32-5p significantly decreased angiogenesis in CCSCI rats; however, the overexpression of miR-32-5p and Xist had no effect on angiogenesis. Furthermore, the overexpression of miR-32-5p inhibited VEGF expression, while the overexpression of miR-32-5p and Xist significantly promoted VEGF expression at 8 wpi (Supplementary Figure S6B). These data suggest that the overexpression of miR-32-5p can inhibit endogenous neurological repair and angiogenesis after CCSCI. Furthermore, we detected the expression levels of Notch-1 and Hes-1 by immunohistochemical staining and observed downregulation in the miR-32-5p group as compared with the miR-32-5p + Xist group (Figures 5G–I). These results demonstrate that miR-32-5p plays a role in endogenous neurological repair and angiogenesis through repression of the Notch-1 pathway by interacting with Xist.

### Xist Promotes Angiogenesis by Regulating the miR-32-5p/Notch-1 Axis *in vitro*

**FIGURE 6:**
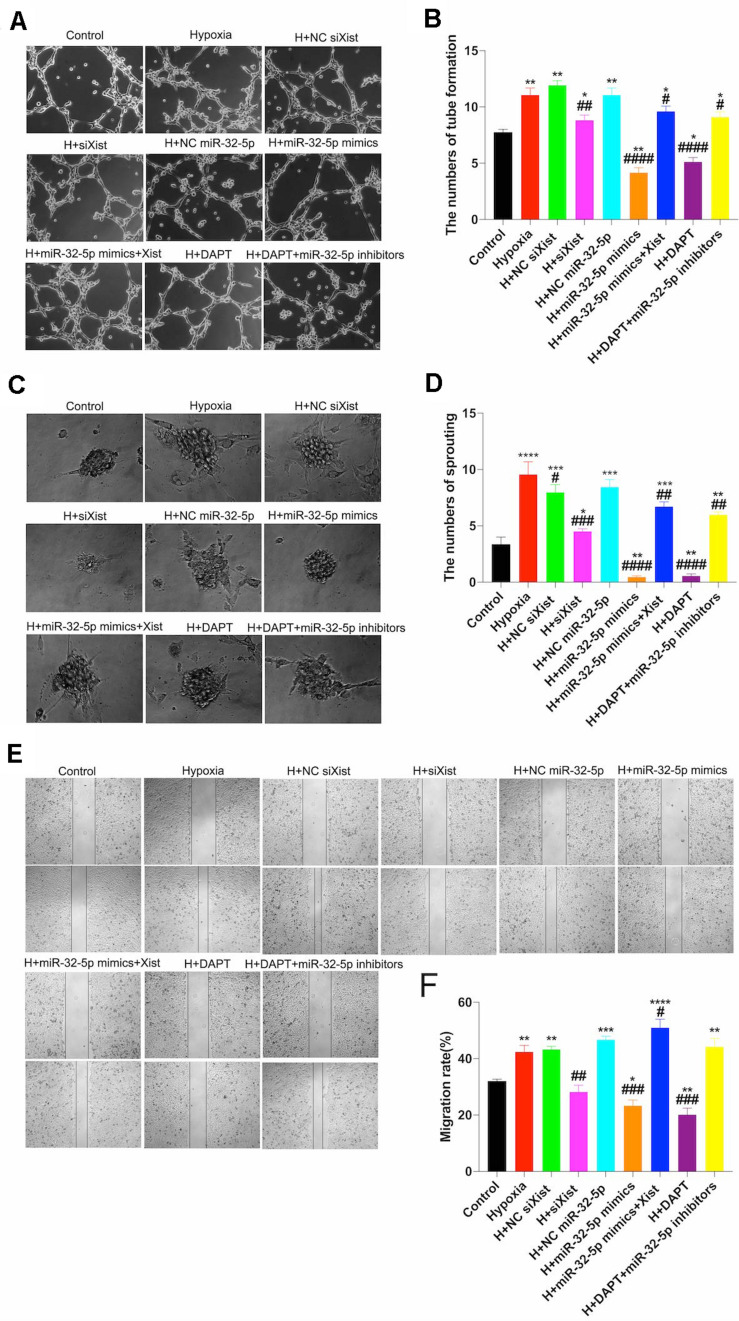
Effect of the Xist/miR-32-5p axis on angiogenesis in HUVECs. **(A)** Representative images of the tube formation assay. HUVECs were exposed to different treatments for 48 h and subsequently subjected to hypoxia for a further 48 h. Tube formation was measured using a CCK8 kit, and sprouting and wound healing assays were performed. **(B)** Quantitation of tube formation was performed by RT-qPCR. **(C)** Representative images of the sprouting assay. **(D)** Quantitation of sprouting was performed by RT-qPCR. **(E)** Representative images of wound healing. **(F)** Quantitation of the migration rate was performed by RT-qPCR. Three independent experiments were carried out. *p < 0.05, **p < 0.01, ***p < 0.001, ****p < 0.0001 compared with the control group; ^#^*p* < 0.05, ^##^*p* < 0.01, ^###^*p* < 0.001, ^####^*p* < 0.0001 compared with the hypoxia group. Data are expressed as the mean ± SEM.

To further understand the regulatory mechanism of the Xist/miR-32-5p/Notch-1 axis, we designed further experiments in cultured HUVECs under normal and hypoxic conditions to simulate the ischemic-hypoxic phase and evaluate tube formation, sprouting, and wound healing (Figures 6A,C,E). Similar to the *in vivo* experiments, we transfected HUVECs with siXist to downregulate the expression level of Xist, and with miR-32-5p mimics or inhibitors to alter the expression level of miR-32-5p. Moreover, we also used DAPT to inhibit the Notch-1 signal pathway *in vitro*. Figure 6 clearly shows that tube formation, sprouting number, and migration rate were increased after 48 h under hypoxic conditions, which is consistent with the results that angiogenesis started at 4 wpi in CCSCI rats. In addition, in comparison with the other groups under hypoxic conditions, HUVECs transfected with siXist or miR-32-5p or treated with DAPT displayed significantly less tube formation and sprouting and a lower migration rate. However, not only did we find that co-transfection with miR-32-5p and Xist significantly promoted angiogenesis in HUVECs but also treatment with DAPT and downregulation of miR-32-5p increased angiogenesis under hypoxic conditions. These results indicate that overexpression of miR-32-5p negatively regulates HUVEC angiogenesis, and inversely, upregulation of Xist and Notch-1 promotes angiogenesis under hypoxic conditions (Figures 6B,D,F).

**FIGURE 7:**
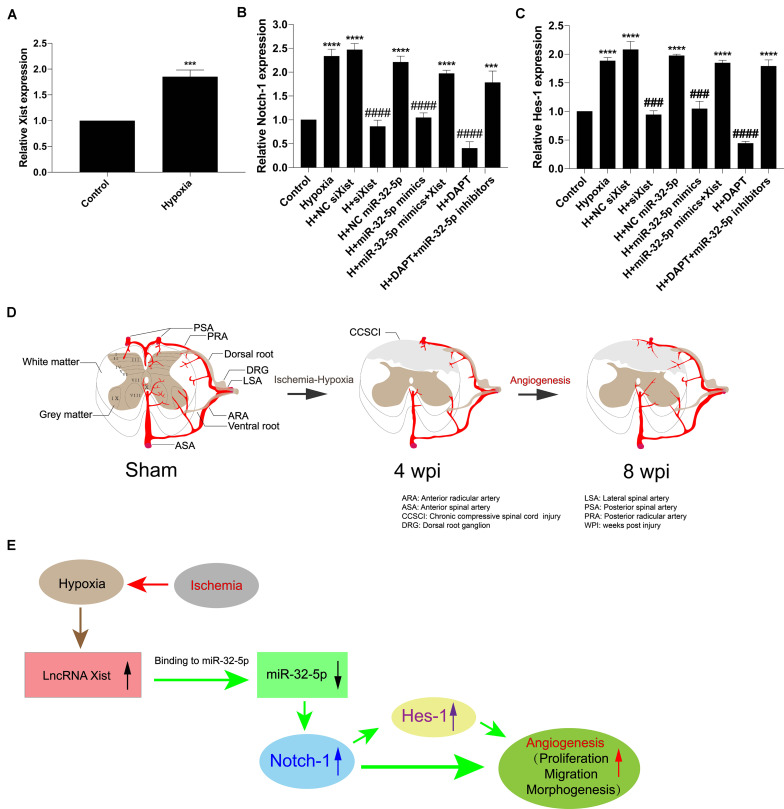
The Xist/miR-32-5p axis exerts its role in angiogenesis by regulating Notch-1 expression. **(A)** The expression of Xist in HUVECs under hypoxic condition was quantitated by RT-qPCR. **(B)** HUVECs were exposed to different treatments. The expression of Notch-1 in HUVECs following transfection was quantitated by RT-qPCR. **(C)** The expression of Hes-1 following transfection was quantitated by RT-qPCR. Three independent experiments were carried out. ***p < 0.001, ****p < 0.0001 compared with the control group; ^###^*p* < 0.001, ^####^*p* < 0.0001 compared with the hypoxia group. Data are expressed as the mean ± SEM. **(D)** Diagram of the angiogenesis process after CCSCI. **(E)** The hypothetical function of Xist in hypoxia-induced angiogenesis in the current study.

In addition, we confirmed that Xist expression was upregulated under hypoxic conditions (Figure 7A). Further, we detected Notch-1 and Hes-1 expression by RT-qPCR to explore whether alteration in the Xist/miR-32-5p axis regulates HUVEC angiogenesis via the Notch-1 pathway. In accordance with the Notch-1 expression pattern in CCSCI rats, Notch-1 (Figure 7B) and Hes-1 (Figure 7C) expression levels were higher in the hypoxia groups (Hypoxia; H + NC siXist; H + NC miR-32-5p; H + miR-32-5p mimics + Xist, and H + DAPT + miR-32-5p inhibitors) as compared with the normoxia groups. However, cells transfected with siXist or miR-32-5p mimics or treated with DAPT showed lower Notch-1 and Hes-1 expression levels than the hypoxia group (Figures 7B,C). These results further confirm that miR-32-5p can repress Notch-1 expression *in vitro*. Collectively, our data imply that lncRNA Xist is involved in endogenous neurological repair and angiogenesis by sponging miR-32-5p, which negatively regulates the expression of Notch-1 (Figures 7D,E).

## Discussion

The present study clearly demonstrates that Xist promoted endogenous neurological repair and angiogenesis after CCSCI. The functional impact of Xist may be through Notch-1, which plays a positive role in enhancing angiogenesis during endogenous neurological repair. Notch-1 and its downstream gene Hes-1 were decreased after depletion of Xist both *in vivo* and *in vitro*. Mechanistically, we found that Xist may act as a competing endogenous RNA (ceRNA) to repress the expression of miR-32-5p. We show that miR-32-5p directly targets Notch-1 to suppress its expression, leading to a reduction in angiogenesis and recessed endogenous neurological repair *in vivo*. In addition, overexpression of Xist sponged miR-32-5p and decreased its expression, improving endogenous neurological repair. Finally, we demonstrate that Xist promoted angiogenesis by regulating the miR-32-5p/Notch-1 axis.

For half a century, Xist has been known as a regulator that transcriptionally silences one of the X chromosomes in mammalian females to achieve dosage compensation. It is surprising to find that Xist can also promote endogenous neurological repair and angiogenesis after CCSCI. However, a significant difference in neuronal recovery between genders has been observed in both patients and animal models. In the last decade, the potential clinical relevance of gender to clinical and neurological outcomes after SCI has attracted much attention (Furlan et al., 2005, 2019a,b; Kay et al., 2010; Chan et al., 2013). With respect to the effects of different genders on locomotor recovery, Datto et al. (2015) reported significant differences in female rats as early as 4 weeks post-SCI, which remained significant at 13 weeks post-SCI. Similarly, others have proposed that the recovery of neurological function in females is often better than in males after SCI in both human and rodent studies (Sipski et al., 2004; Xiao et al., 2017). However, the underlying molecular mechanism for the gender difference in neurological function recovery has not yet been elucidated. In combination with previous observations from clinical and rodent studies, our findings provide new insight to explain the gender differences regarding endogenous neurological repair after CCSCI.

We propose that Xist contributes to endogenous spinal cord repair through the regulation of Notch-1 by competing for miR-32-5p. We identified that Xist and Notch-1 are targets of miR-32-5p by bioinformatics prediction and validated the regression effect of miR-32-5p using *in vitro* experiments. We found that miR-32-5p overexpression can decrease Notch-1, and co-transfection with Xist can offset the negative biological effects resulting from overexpression of miR-32-5p. Canonically, Xist is localized in the nucleus, while miRNAs are typically found in the cytoplasm. One mystery remains: the location at which Xist sponges miR-32-5p. Many studies have shown that Xist can act as an miRNA sponge in other diseases: Wei et al. (2018) suggested that Xist contributes to neuropathic pain development by sponging miR-154-5p in rats with chronic constriction injury; Cheng et al. (2017) reported that Xist can promote glioma angiogenesis by acting as a molecular sponge of miR-429; Yu et al. (2017) showed that depletion of Xist is able to inhibit glioma angiogenesis and increase blood-tumor barrier permeability by targeting miR-137; and Hu et al. (2019) suggested that Xist participates in hypoxia-induced angiogenesis in human brain microvascular endothelial cells by regulating the miR-485/SOX7 axis. According to this evidence, we hypothesized that Xist may be released from the nucleus and act as a sponge for miR-32-5p in our scenario. Xist has indeed been shown to be present throughout the cytoplasm by RNA-FISH and other techniques (Cabili et al., 2015; Carlevaro-Fita et al., 2016). Taken together, these data indicate that Xist plays an important role in the pathophysiology of ischemia-related diseases with respect to its basic molecular regulatory function.

In our previous studies, we have reported that angiogenesis is related to the recovery of neurological function (Long et al., 2014; Cheng et al., 2015). Here, we confirm that angiogenesis serves as a beneficial factor to improve neurological function after CCSCI, which is supported by many other published studies. For instance, the ubiquitously transcribed tetratricopeptide repeat on chromosome X (UTX)/lysine demethylase 6A (KDM6A) deletion in a mouse SCI model has been shown to promote neurological function by epigenetically regulating vascular regeneration (Ni et al., 2019). Moreover, hepatocyte growth factor was demonstrated to enhance endogenous repair and functional recovery in a rat SCI model by promoting neuron and oligodendrocyte survival, angiogenesis, and axonal regrowth (Kitamura et al., 2007). In the present study, we further found that Xist overexpression can improve neurological endogenous repair by promoting endogenous angiogenesis after CCSCI. Activation of the Notch-1 pathway, an important regulator of angiogenesis, is necessary for the process of endogenous neurological repair.

The current study also has several limitations. First, intraperitoneal/intravenous injection has been applied in nervous system studies as a systemic delivery method (Machida et al., 2013; Njoo et al., 2014). We also revealed that Xist expression was inhibited after Xist knockdown via systemic delivery methods. Intrathecal injection has shown benefits over other systemic delivery routes. These benefits include more specific spinal modulation, which permits reduced dosages and limits side effects. Technically, whether an intraperitoneal/intravenous delivery method can also result in side effects in other systems remains unclear. The second limitation is that ischemia-hypoxia changes were observed after CCSCI. However, more experiments are needed to further illuminate how hypoxia induces the upregulation of Xist after CCSCI. Thirdly, it remains unclear whether a different direction of the implant in the spinal cord may lead to compressive injury in a different spinal cord area or tract, thus resulting in different motor performance.

## Conclusion

The present study indicates a positive role for the lncRNA Xist in endogenous spinal cord repair following CCSCI. We show that Xist can promote endogenous repair and facilitate angiogenesis by acting as an miR-32-5p/Notch-1 axis regulator. Our findings suggest that Xist may act as a novel molecular therapeutic target for CCSCI and provide new insight into the gender differences in CCSCI.

## Data Availability Statement

The original contributions presented in the study are included in the supplementary material, further inquiries can be directed to the corresponding author.

## Ethics Statement

The animal study was reviewed and approved by The Research Ethics Committee of Sun Yat-sen University, Guangzhou, China.

## Author Contributions

XC performed the experiments, analyzed the data, and wrote the manuscript. JX conceived the study and revised the manuscript. ZY and JHX performed the experiments and analyzed the data. HL conceived, designed, and supervised the research. All authors read and approved the final manuscript.

## Conflict of Interest

The authors declare that the research was conducted in the absence of any commercial or financial relationships that could be construed as a potential conflict of interest.
