# Supplementary material for: LncRNA Xist Contributes to Endogenous Neurological Repair After Chronic Compressive Spinal Cord Injury by Promoting Angiogenesis Through the miR-32-5p/Notch-1 Axis
Source: Front Cell Dev Biol. 2020 Aug 6;8:744. doi: 10.3389/fcell.2020.00744 (PMC7423840; doi:10.3389/fcell.2020.00744)
Supplement: Supplementary file 1 [file Data_Sheet_1.docx]

**Supplemental Information**

**Table S1. The sequences of siXist and NC siXist**

| Gene | Sequence |
| --- | --- |
| siXist | 5'-TGTCTCTTTCTTTCTTGTCTTTGCT-3' |
| NC siXist | 5'-TCTATCTAGTAAATTCTGCCGTCAT-3' |
| miR-32-5p mimics | 5'-UAUUGCACAUUACUAAGUUGCA-3' |
| miR-32-5p inhibitors | 5'-UGCAACUUAGUAAUGUGCAAUA-3' |
| NC miR-32-5p | 5'-UCACAACCUCCUAGAAAGAGUAGA-3' |

**Table S2. Primers used for RT-qPCR**

| Gene | Forward primer (5'-3') | Reverse primer (5'-3') |
| --- | --- | --- |
| Xist | CAGACGTGTGCTCTTC | CGATCTGTAAGTCCACCA |
| miR-32-5p | CCGACAACCACTACCTGA | ATTGTGGATGAATACTGCC |
| U6 | CTCGCTTCGGCAGCACA | AACGCTTCACGAATTTGCGT |
| GAPDH | CAAGGTCATCCATGACAACTTTG | GTCCACCACCCTGTTGCTGTAG |
| Notch-1 | CTGGTCAGGGAAATCGTG | TGGGCAGTGGCAGATGTAG |
| Hes-1  HIF-1α | ACACCGGACAAACCAAAGAC  TATGAGCCAGAAGAACTTTTAGGC | AATGCCGGGAGCTATCTTTC  CACCTCTTTTGGCAAGCATCCTG |


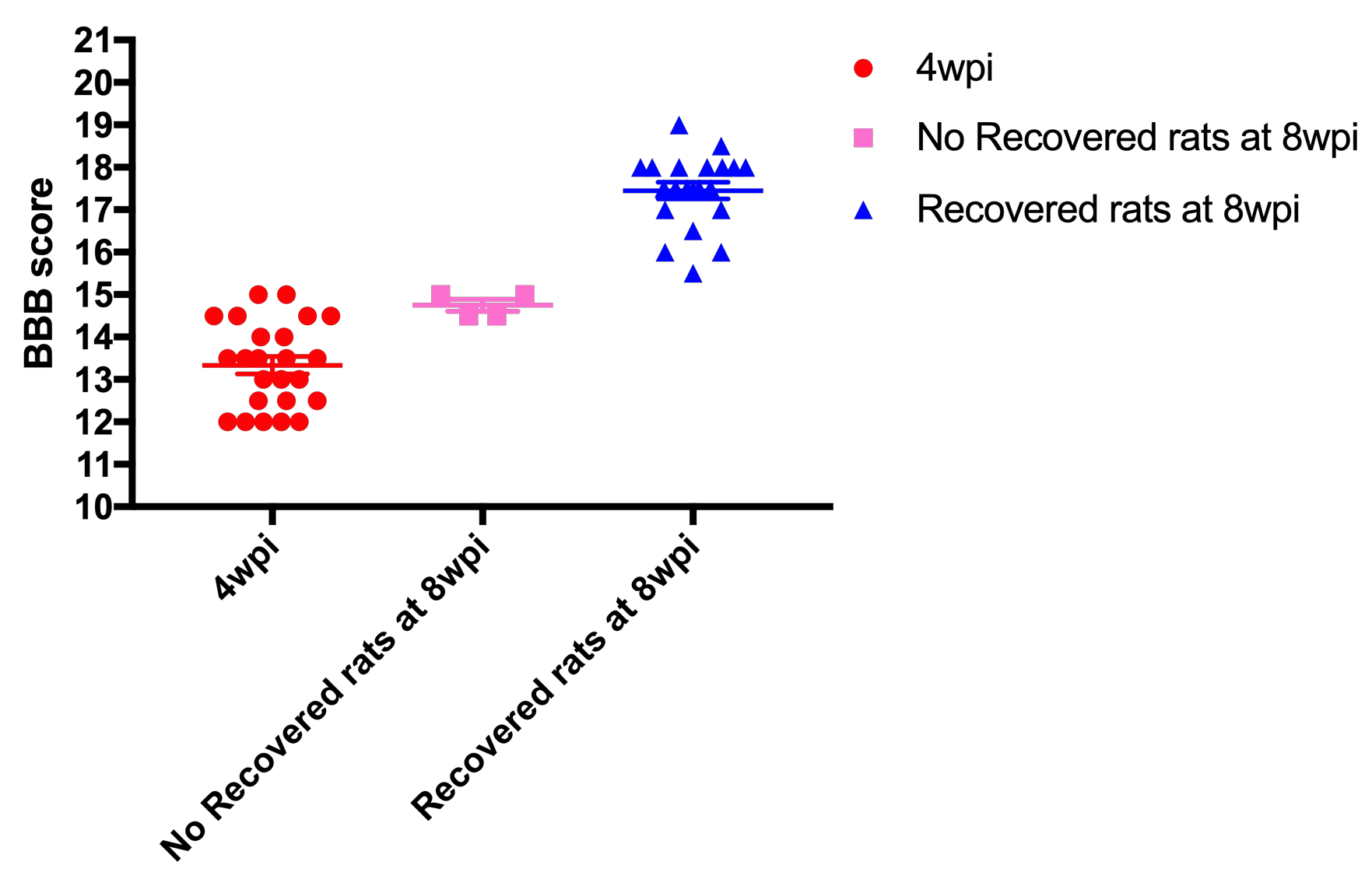


**Figure S1. BBB score for recovered and unrecovered rats at 8 wpi.**

The BBB score at 4wpi after CCSCI showed small variations among rats. At 8 wpi, the recovered rats and unrecovered rats show different BBB scores. Four of the twenty-four animals in the 8W SCI group did not significantly recover at 8 wpi according to the BBB score. Data are expressed as the mean ± SEM.


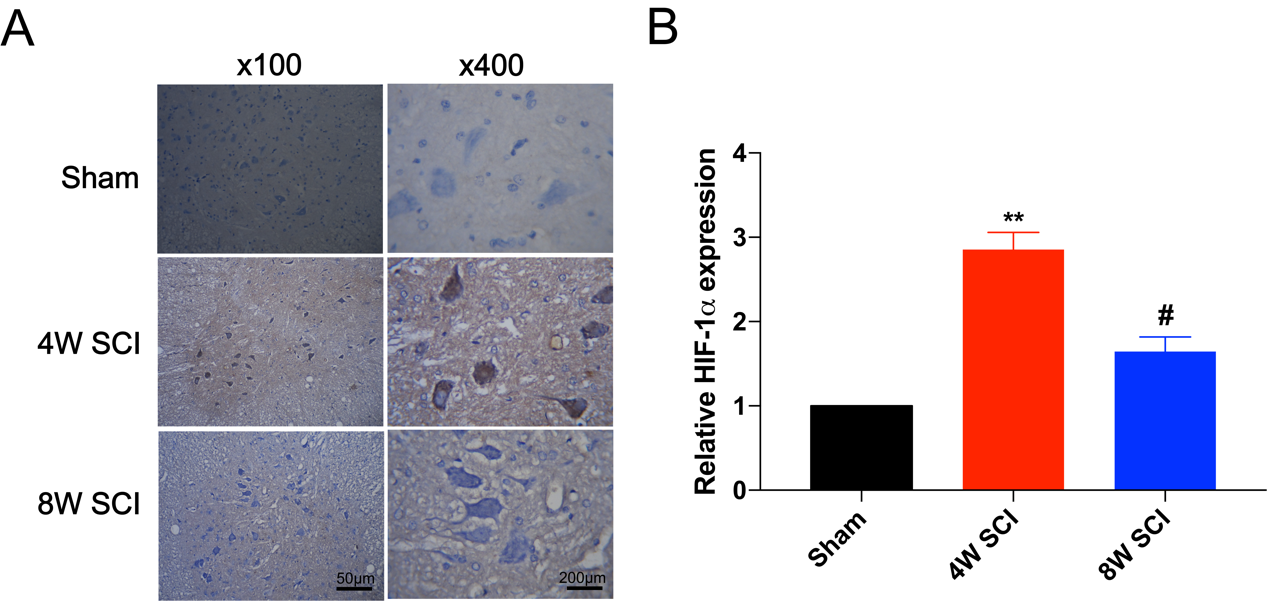


**Figure S2.** **HIF-1α expression after CCSCI.**

(A) ISH of HIF-1α mRNA: almost no HIF-1α expression was detected in sham animals; marked HIF-1α expression was detected in the 4W SCI group; and significantly decreased HIF-1α expression was detected in the 8W SCI group (100× magnification: scale bar, 50 μm; 400× magnification: scale bar, 200 μm). (B) Quantitation of relative HIF-1α expression by RT-qPCR. ***p* < 0.01 compared with the sham group; ^#^*p* < 0.05 compared with the 4W SCI group. Data are expressed as the mean ± SEM.


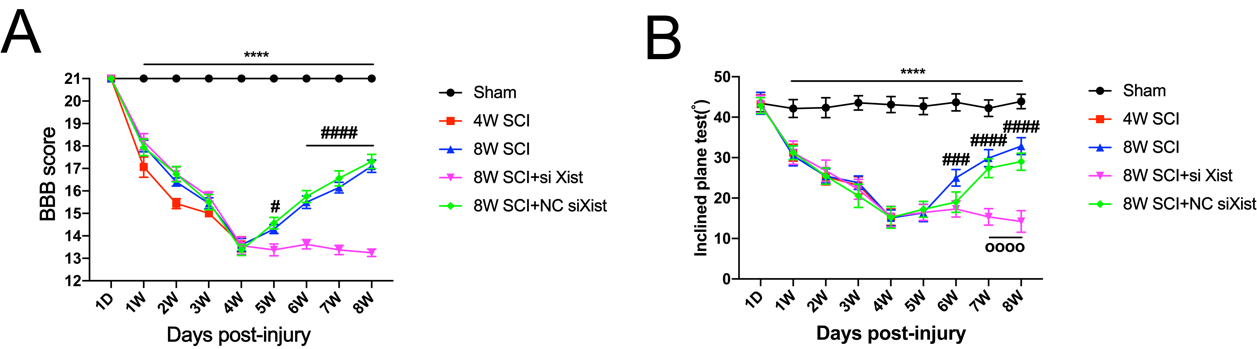


**Figure S3. The knockdown of Xist inhibited endogenous neurological repair after CCSCI.**

(A) The neurological function was evaluated using the BBB score. *****p* < 0.0001 the sham group vs. the 8W SCI group; ^#^*p* < 0.05, ^####^*p* < 0.0001 the 8W SCI+siXist group vs. the 8W SCI+NC siXist group. (B) The neurological function was evaluated using IPT. *****p* < 0.0001 the sham group vs. the 8W SCI group; ^###^*p* < 0.001, ^####^*p* < 0.0001 the 8W SCI+siXist group vs. the 8W SCI group. ^oooo^*p* < 0.0001 the 8W SCI+siXist group vs. the 8W SCI+NC siXist group. Data are expressed as the mean ± SEM.


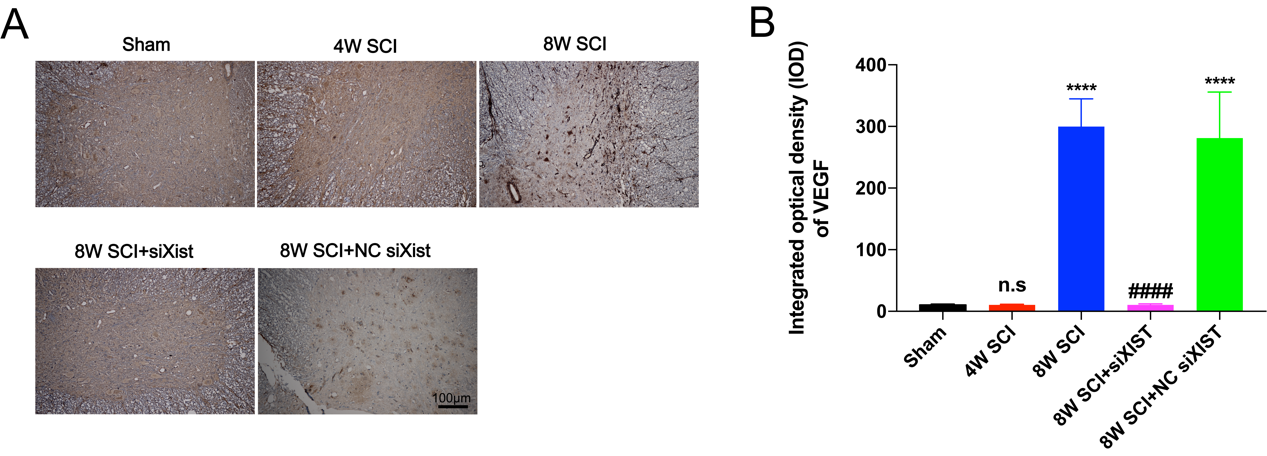


**Figure S4. The knockdown of Xist inhibited angiogenesis after CCSCI.**

(A) Angiogenesis was assessed by VEGF immunostaining (200× magnification; scale bar, 100 μm); representative images are shown. VEGF was mainly expressed in the ventral horn of the cervical spinal cord. (B) VEGF staining was quantitated by analyzing the integrated optical density (IOD). n.s (non-significant) compared with the sham group; *****p* < 0.0001 compared with the 4W SCI group; ^####^*p* < 0.0001 compared with the 8W SCI group. All data are expressed as the mean ± SEM.


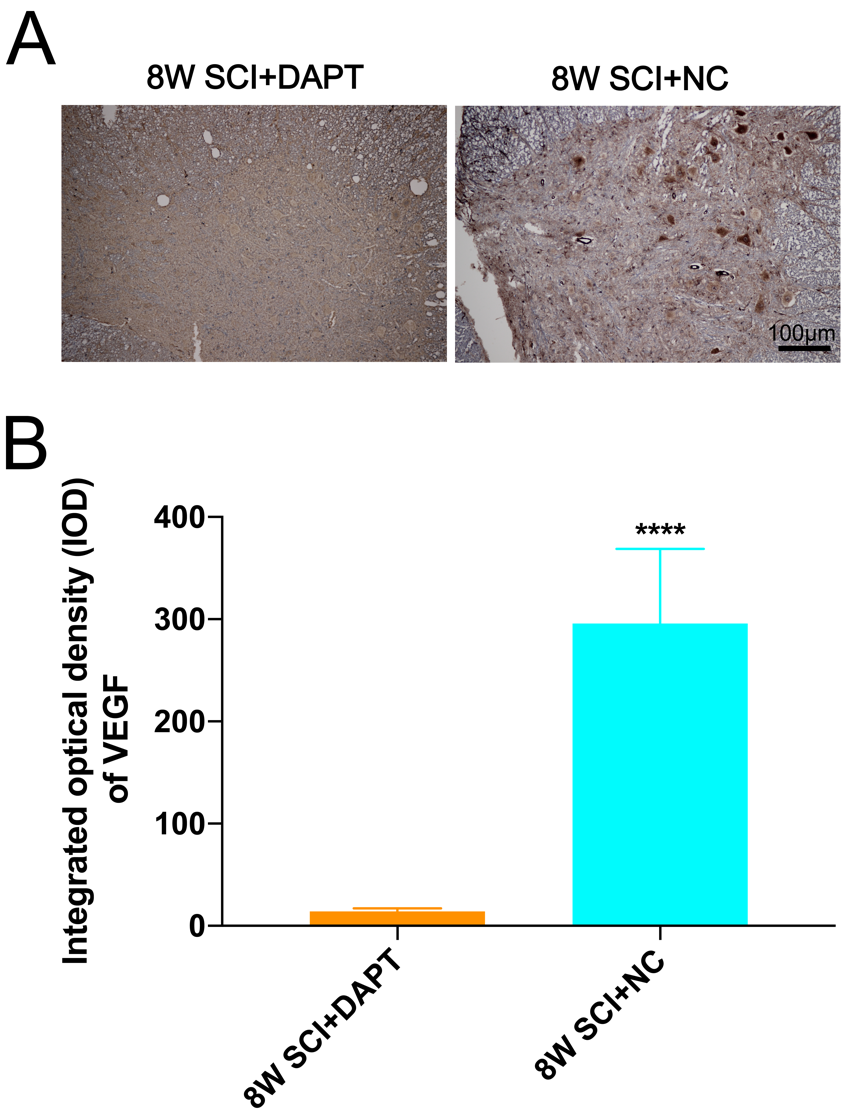


**Figure S5. Notch-1 inhibited angiogenesis after CCSCI.**

(A) Angiogenesis was assessed by VEGF immunostaining (200× magnification; scale bar, 100 μm); representative images are shown. (B) VEGF staining was quantitated by analyzing the integrated optical density (IOD). *****p* < 0.0001. All data are expressed as the mean ± SEM.


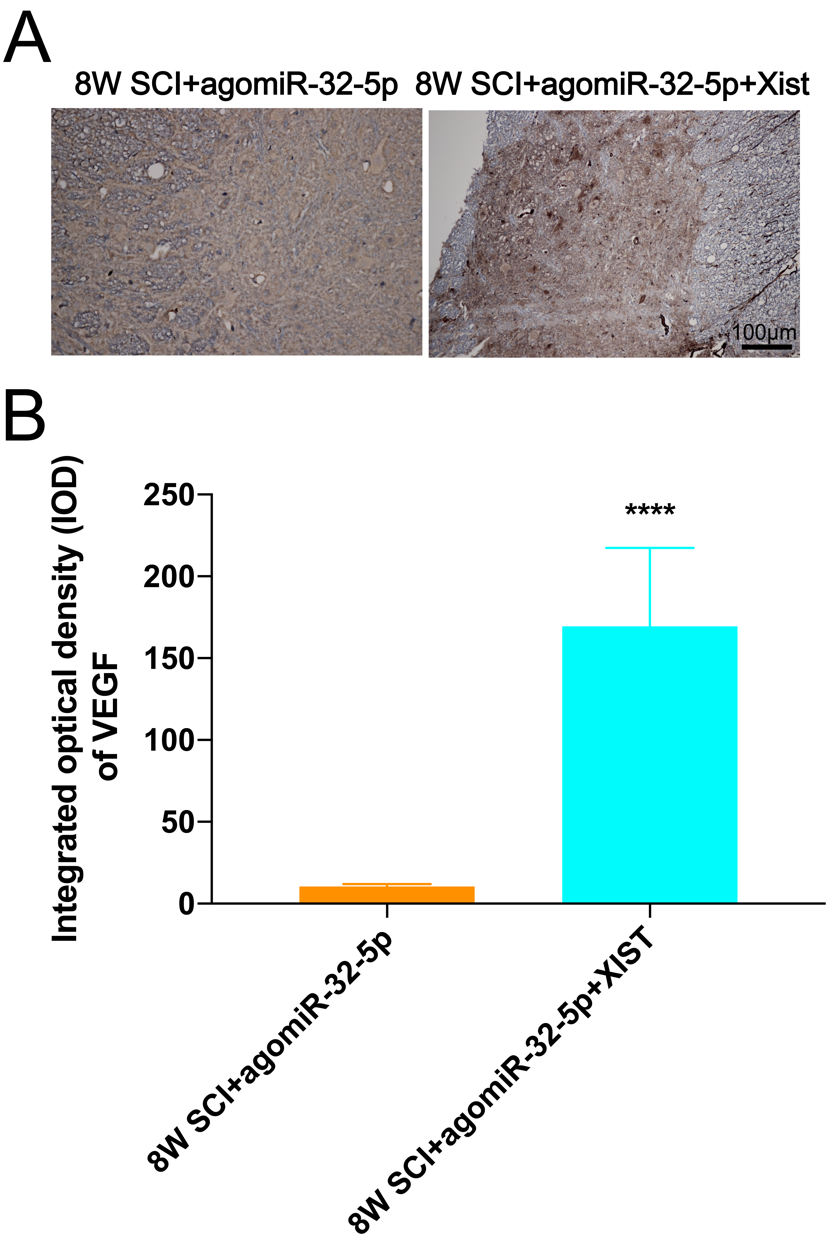


**Figure S6. Overexpression of miR-32-5p inhibited angiogenesis after CCSCI.**

(A) Angiogenesis was assessed by VEGF immunostaining (200× magnification; scale bar, 100 μm); representative images are shown. (B) VEGF staining was quantitated by analyzing the integrated optical density (IOD). *****p* < 0.0001. All data are expressed as the mean ± SEM.
